# Smartphone Acoustic Sensing for Contactless Respiration Monitoring and Gesture-Based Authentication

**DOI:** 10.3390/s26175512

**Published:** 2026-08-31

**Authors:** Raj Nandini, Ashish Mishra

**Affiliations:** 1 Department of Electrical Engineering and Computer Science, Syracuse University, Syracuse, NY 13244, USA; 2 Independent Researcher, Irvine, CA 92618, USA

**Keywords:** acoustic signals, Doppler, envelope detection, handheld sonar, ultrasonic

## Abstract

This paper presents a smartphone-based sonar-sensing system for contactless monitoring of respiration and gesture-based user activity, using a single commercial phone with no hardware modification. A 20 kHz audio signal is transmitted from the phone’s speaker and reflections are captured by its microphone; motion-induced Doppler shifts are recovered using an envelope detection method that removes the need for a reference copy of the transmitted signal and improves robustness to transmitter frequency drift. Actuator experiments at a target distance around 50 cm demonstrated motion sensitivity at two conditions, 6 mm at 0.15 Hz and 2 mm at 0.6 Hz, with a measured signal-to-noise ratio of approximately 21.7 dB. Respiration rate detected by the system was compared against a pulse oximeter across two subjects, with an error within 0.1 Hz of the reference. Gesture-based interactions (tapping, scrolling, typing, talking) were analyzed using short-time Fourier transform features as a first step toward device authentication. Distance and direction estimation are outside the scope of this work and are identified as directions for future development.

## 1. Introduction

Remote measurement techniques have generated a lot of interest in recent years, including camera-based measurement systems, infrared sensors, radar, lidar, and ultrasonic sensor/sonar.

The camera-based measurement systems [1,2,3] have evolved a lot, and their accuracy has been improved significantly. However, their measurements are affected by the lighting conditions of the room, and people are not inclined to use camera-based sensors due to privacy concerns.

The infrared sensors [4] provide secure communication due to the line-of-sight mode of communication. Their small size, high stability, and low cost make them an ideal candidate for remote measurement; however, they suffer from multiple disadvantages, such as high power from infrared radiation that can damage eyes, unreliability in measuring large distances, and the susceptibility of readings to errors caused by smoke, dust, fog, and sunlight.

The radar [5,6,7,8,9,10] has been used for a long time for an accurate measurement of target motion. They have found use in health care, localization, and many more cases. However, radars operate using electromagnetic waves, and although typical low-power sensing radars operate well within established exposure safety limits, public perception of RF radiation exposure remains a barrier to adoption in some consumer and healthcare contexts [11].

Lidars [12,13,14] are one of the safest, fastest, and most accurate methods of localizing the target. However, they suffer from high cost and an extremely large volume of data. This high cost and large data size do not make them an attractive choice for mass deployment as everyday monitoring equipment.

The next most used for remote sensing are sonars [15,16,17]. Here, sound waves are transmitted and received to determine the motion and range of the target. Since sound waves are used, they do not have an unfavorable impact on humans. The cost of the sonar is lower than the cost of a lidar. All these make the sonar an ideal choice for mass deployment devices for human vital signs and gesture measurement. 

In ref. [18], two mobile phones were utilized. One phone was used as a transmitter, while the other phone was used as a receiver. The received audio file containing the Doppler frequency of the target was segmented, and FFT was performed to determine the motion of the target for each segment. The above approach requires at least two phones, i.e., one phone for the transmitter and the other phone as a receiver. This limits the potential applications of the above approach that will be difficult to implement in the real-world scenario, i.e., walking on the street.

In the works [19,20], for extracting the Doppler frequency from the received signal, sin ft and cos ft were multiplied. Here, the two signals were computer-generated. This method works if the transmitting signal does not drift and is always equal to f. However, if there is a drift in the transmitted frequency, or if the transmitted frequency is different from f even by a few hertz, the above approach does not yield the correct result.

The work [21] utilizes the frequency shift detection model to detect the motion. Here, the received signal was segmented into smaller parts, and wavelets were performed on it. A Butterworth bandpass filter was also introduced to filter out the out-of-band interferences. Utilizing filters to filter out unwanted signals might also result in filtering out useful data. The approach discussed here limits the application of the system. Utilizing segmentation and wavelet approach may prevent real-time operation.

Utilizing the down-conversion using multiplication approach without the transmitted copy of the signal, i.e., by multiplying the received signal with sin 2πft and cos 2πft where f = 20 kHz, results in the baseband data, which contain the motion of the target. However, if there is a drift in the transmitted audio signal, the above approach fails and does not provide the correct result. In this paper, the down-conversion of the received signal was performed using the envelope approach. The upper and lower envelopes of the signals were utilized in the FFT calculation. Since the positive and negative frequency components are mirror images of each other, only a positive portion is used. Table 1 contains the comparison of the proposed work with a state-of-the-art model.

Existing smartphone acoustic-sensing approaches for Doppler-based motion detection generally rely on down-conversion by multiplying the received signal with sin(2πft) and cos(2πft) at the known transmitted frequency f [19,20]. This approach assumes the transmitted frequency remains exactly equal to f throughout the measurement. In practice, consumer smartphone audio hardware exhibits frequency drift due to oscillator tolerance and thermal effects, and even a drift of a few hertz causes the down-conversion to fail, since the multiplication no longer isolates the correct baseband component. An alternative approach segments the received signal and applies bandpass filtering with wavelet analysis [21], but filtering risks removing genuine motion-related signal along with noise, and segmentation-based processing is not well suited to real-time operation. The envelope-based method proposed here avoids both limitations: it requires no reference copy of the transmitted signal and no assumption that the transmitted frequency is precisely known, and it operates directly on the recorded waveform without segmentation, preserving compatibility with real-time processing.

## 2. System Operation in Doppler Mode and Algorithm

The mobile phone was used as a sonar device where the sound wave at 20 kHz was radiated into the environment using the phone’s speaker, and the reflected signal was captured by the microphone of the mobile phone adjacent to it. In a normal scenario, the speaker and the microphone do not work simultaneously. Hence, to solve this issue, a mobile application (app) was used that can control the speaker and record data simultaneously. The app transfers the data in the local drive to the computer, where it is processed by the algorithms to derive signals of interest.

**Figure 1:**
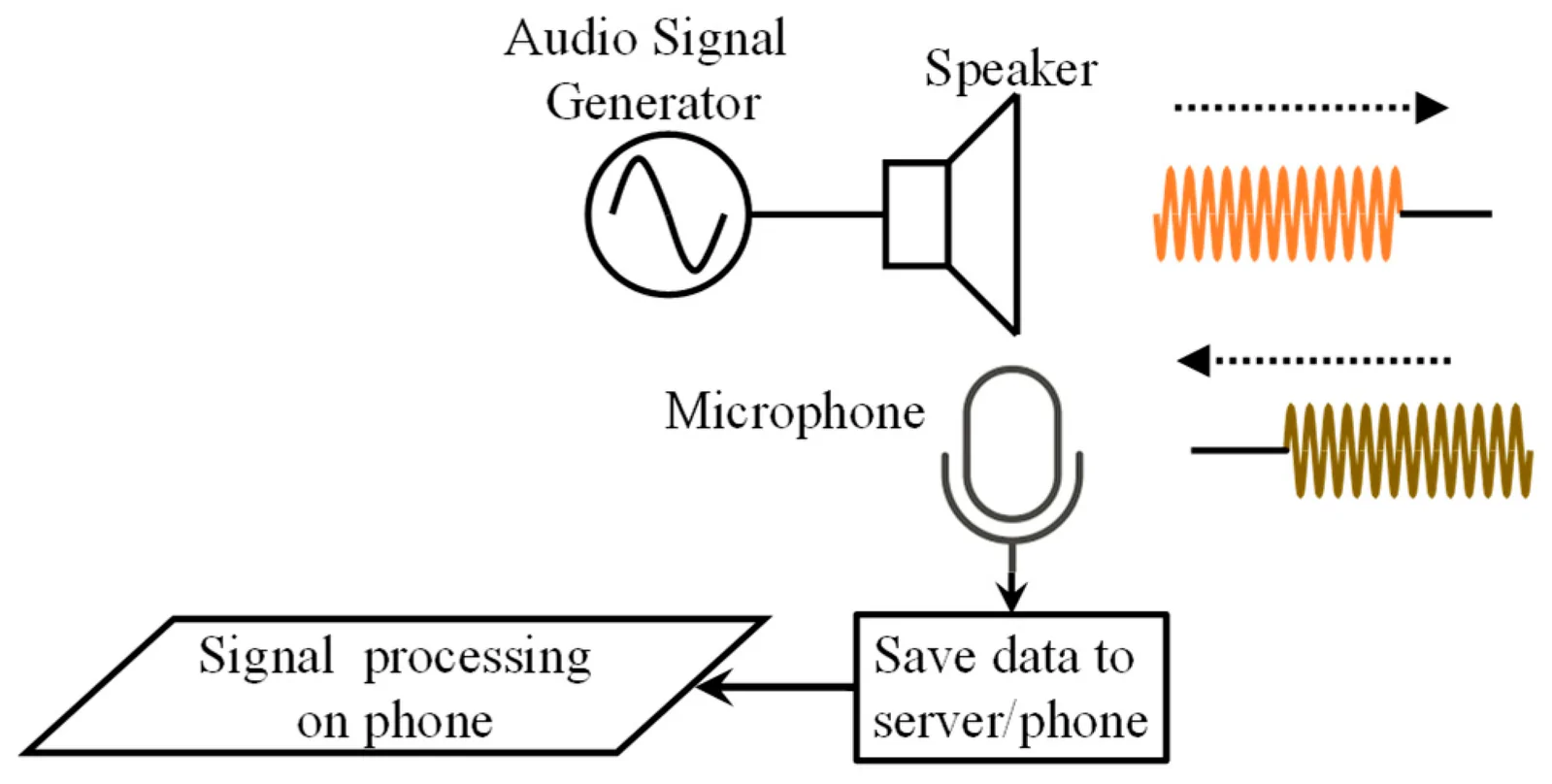
Block diagram showing the transmission and receiving of soundwaves, data acquisition and processing of the waveform.

Smartphones have a high-pass filter at the microphone input that restricts recording of frequencies below 20 Hz. In order to record motion below 20 Hz, the motion signal should be added to a carrier audio signal with frequency above 20 Hz so that this modulated signal can pass through this filter. In this work, a 20 kHz carrier was used since higher-frequency signals are relatively silent to the human ear and do not cause disturbance to human beings. Figure 1 shows the overview of the entire sensing operation.

The received signal contains the transmitted signal modulated by the motion frequency of the target. The signal was recorded with a sampling frequency of 44.1 kHz. In order to correctly record the data, the sampling frequency is selected for at least twice the frequency of interest.

The transmitted signal from the phone is expressed as

(1) $$T\left(t\right)=\cos\left[2\pi f_{t}t+\phi \left(t\right)\right]$$

where $f_{t}$ is the transmitting frequency and *ϕ*(*t*) is the phase noise of the signal generator. The transmitted frequency is sent to a distance $x_{o}$, which is modulated by the movement of the target.

(2) $$R\left(t\right)=\cos\left[2\pi f_{t}t-\frac{4\pi x_{o}}{\lambda }-\frac{4\pi x\left(t\right)}{\lambda }-\phi \left(t-\frac{2x_{o}}{c}\right)\right]$$

In Equation (2) the wavelength corresponding to $f_{t}$, $x\left(t\right)$ was the mechanical displacement of the target, while $x_{o}$ was the nominal distance between the target and the sonar. Envelope detection recovers the baseband signal by extracting the slowly varying amplitude modulation of *R*(*t*) while discarding the fast oscillation at the carrier frequency $f_{t}$. Since $x_{o}$ is constant over the measurement window, it contributes only a fixed phase offset in the argument of *R*(*t*) and does not affect the frequency content of the recovered envelope; it would be relevant only for absolute range recovery, which is not the focus of this work. The residual phase noise term is evaluated at a delay corresponding to the round-trip propagation time 2xoc, and since this delay is small and the oscillator phase noise varies slowly, Δ*φ*(*t*) remains small relative to the motion-induced phase term. The resulting baseband signal, expressed in Equation (3), therefore depends primarily on x(t), the time-varying displacement of the target. 

The baseband signal $B\left(t\right)$ is obtained from the received signal $R\left(t\right)$ utilizing the envelope detection method. The upper and the lower envelope are phase-shifted by 180°. $B\left(t\right)$ can be expressed as

(3) $$B\left(t\right)=\cos\left[\frac{4\pi x(t)}{\lambda }+\Delta \phi (t)\right]$$

where $∆\phi \left(t\right)$ is the residual phase noise from the oscillator, which is very small.

Under normal conditions, either the speaker or the microphone is operational at any given instant. However, this app overrides this feature and makes both the recording and transmission simultaneous. This is one of the most prominent features of the app, which makes it possible for a single mobile phone to operate as sonar.

**Figure 2:**
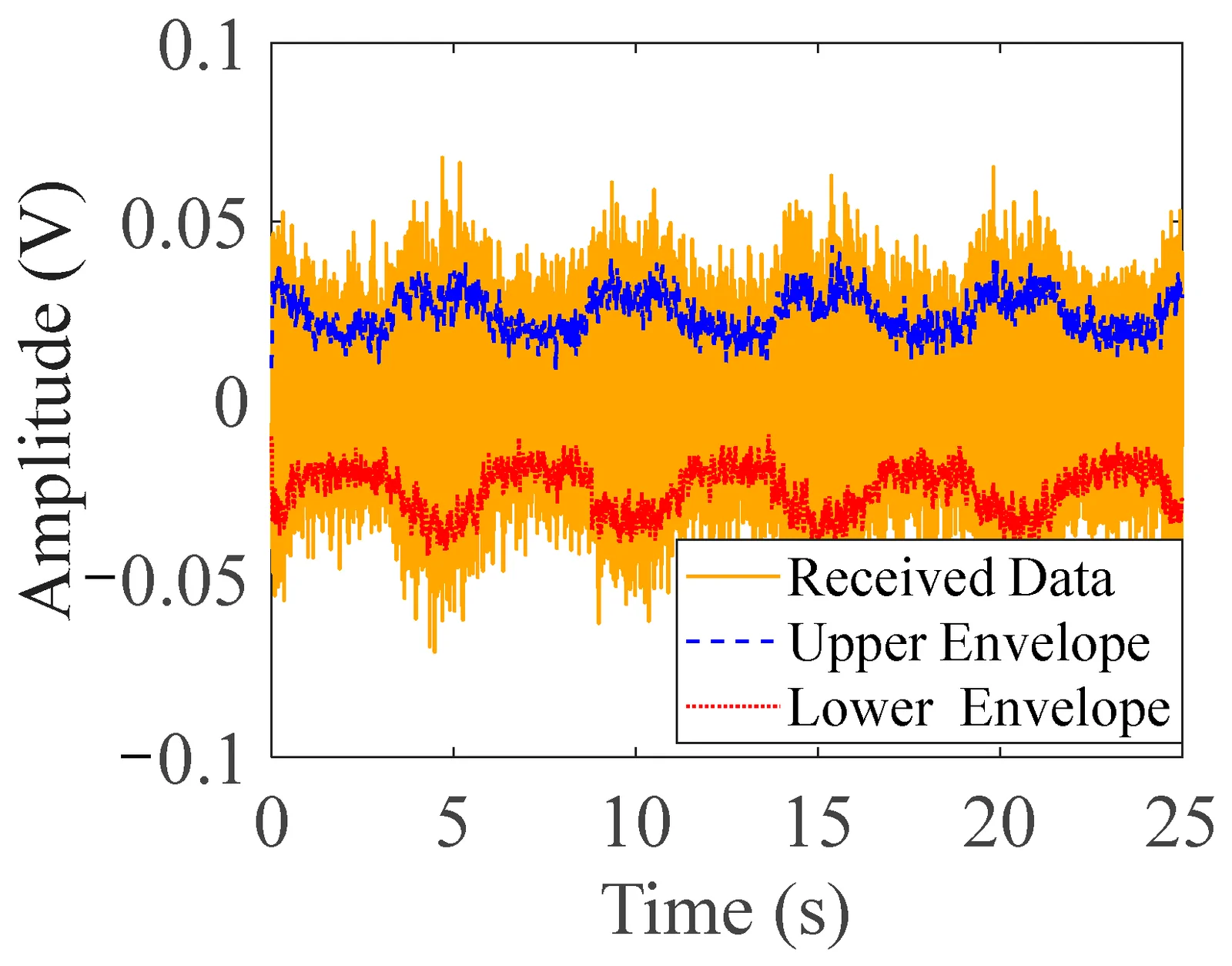
Received data from mobile, upper, and lower envelope data.

Figure 2 shows a recorded received signal obtained by the microphone during measurements and the corresponding envelope of this signal, which contains the information on the target’s motion.

An FFT is performed on the envelope data, and corresponding motion frequency is obtained. To evaluate the robustness of the method, STFT was also performed with a Kaiser window of 10 s and an overlap of 9.5 s on a 28 s data. The proposed system requires a minimum of 10 s of accumulated data to compute a vital-sign estimate, consistent with the window size selected to ensure at least one full respiration cycle is captured (Section 4). Once this window of data is available, the FFT computation itself is computationally lightweight, with FFT/STFT operations on data of this length completing on the order of milliseconds on standard consumer hardware. The system therefore operates in a near-real-time streaming fashion for vital sign detection, where total latency is dominated by the required observation window rather than by computational cost. For gesture detection, which uses a smaller 1–2 s window (Section 4), the corresponding latency floor is proportionally lower, enabling faster response for gesture-based interactions.

## 3. Measurements and Results

**Figure 3:**
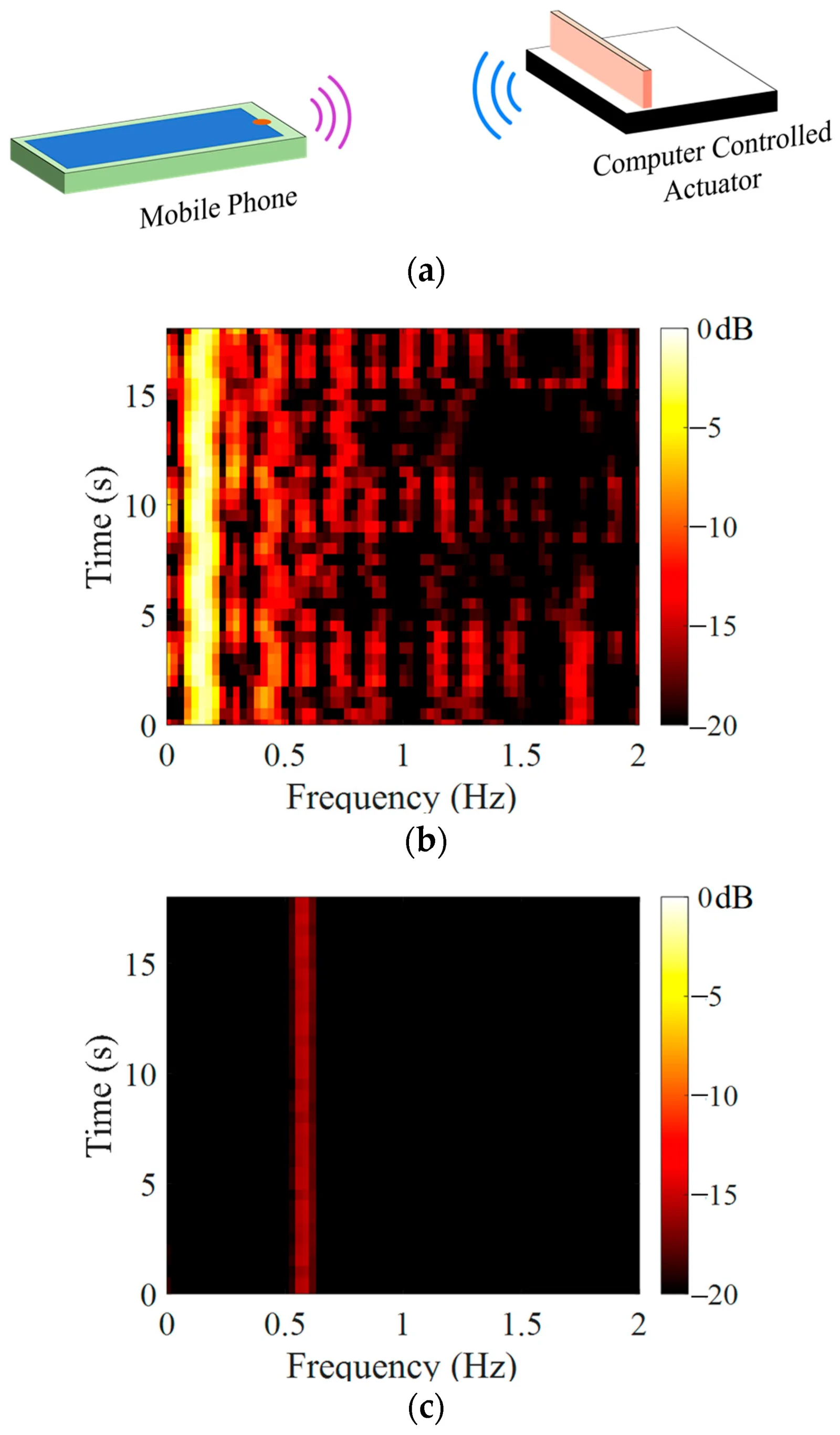
(**a**) Measurement setup for Experiment 1; (**b**) STFT plot for 6 mm at 0.15 Hz; (**c**) STFT plot for 2 mm at 0.6 Hz.

In the experiment, a mechanical motion is produced with the actuator. The actuator used for this experiment was a precision computer-controlled linear micro-actuator, and its motion amplitude and frequency can be programmed with very high accuracy [8]. The experiment was conducted in a typical lab environment, and the actuator and phone were placed on a table, and the measurements were recorded for two conditions: 6 mm at 0.15 Hz, and the other at 2 mm at 0.6 Hz. Figure 3a shows the measurement setup. The mobile phone used for the experiment was NUU S5702L. During our experiments, we have noticed that volume settings have an impact on the received quality of the sound signals. Therefore, the volume was set at approximately 80% of the maximum volume.

The audio signal was generated at 20 kHz and radiated towards the target. The target reflected the audio signal with the motion of the target added to it. The baseband signal was recovered using the envelope detection method. Figure 3b shows the short-time Fourier transform (STFT) plot for a 6 mm motion amplitude at 0.15 Hz, and Figure 3c shows the STFT plot for a 2 mm motion at 0.6 Hz. These two conditions were selected to demonstrate feasibility of the proposed envelope-based method across a representative displacement and frequency combination, rather than to characterize a general displacement sensitivity curve across a range of amplitudes and frequencies. A systematic evaluation over a broader range of displacement amplitudes and frequencies, with associated error, standard deviation, and confidence intervals, is identified as a direction for future work.

## 4. Experiments with Human Subjects

Here, we constructed several experiments (tasks) to collect datasets from end-users for the following three cases: (a) Human absence; (b) Human presence but no interaction with the phone (when the phone was kept on the palm, and when the phone was kept on the table); (c) Human presence and interaction (tapping, scrolling, typing, speaking, and walking).

The gesture experiments involved two participants, with each of the four gesture types (tapping, scrolling, typing, talking) performed 10 times per participant, yielding a total of 20 recordings per gesture across all participants. All gestures were performed by the same two participants, and all recordings were collected within multiple sessions on the same day, in a standard room environment. Feature extraction and STFT analysis were performed with knowledge of the intended gesture for each recording, since the purpose of this analysis was to characterize the STFT signature associated with each known gesture type, rather than to perform blind classification. As noted above, formal blind classification with train/test separation is identified as future work.

The transmitted frequency was set at 20 kHz, and data were recorded at 44.1 kHz sampling frequency to be within the Nyquist limit. The data were recorded for a period of 30 s. However, during processing, the first 2 s of data are discarded to remove a cold-start effect observed at the beginning of each measurement, during which the transmitted and received signal had not yet reached a steady state. Currently, we are accessing the whole audio signal and processing it remotely; this may have some privacy concerns from the user end. To overcome this issue, future work will focus on performing preprocessing on the user’s device and collect only the envelope data. These data will not contain speech information, only the motion information necessary for authentication purposes.

### 4.1. Algorithm

**Figure 4:**
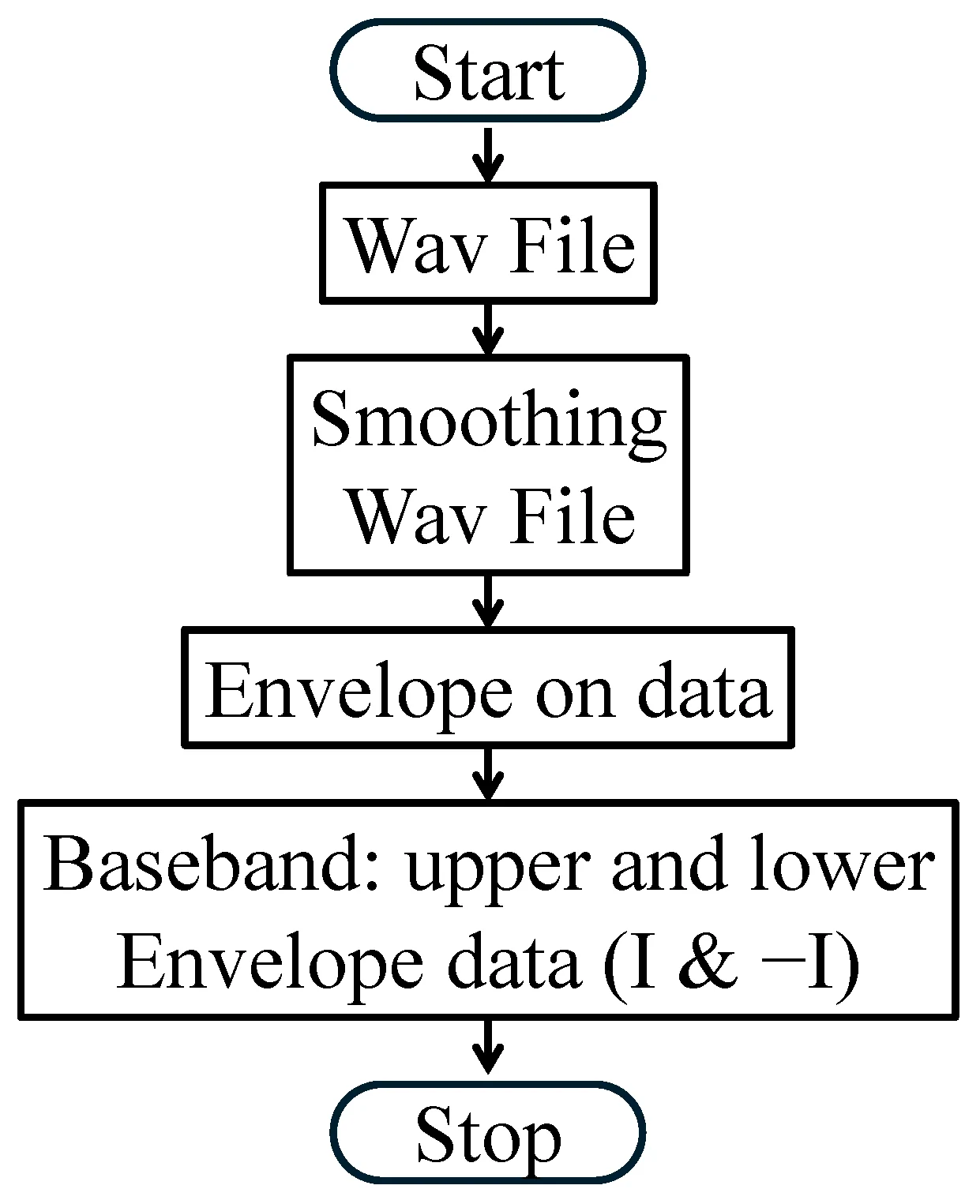
Preprocessing of data.

Pre-processing: The data recorded from the mobile phone were recorded in wav format, and the first 2 s of data were removed and sent to the smoothing stage in the pre-processing algorithm. Here, a moving average of data for every 5 points was applied, and the smoothed data were sent to the envelope extraction stage to extract the Doppler information from the received signal. Figure 4 shows the preprocessing algorithm performed. The normal breathing rate of a human typically lies between 10–20 breaths per minute. This corresponds to a Doppler shift ranging from 0.1 Hz to 0.4 Hz. The Doppler shift due to gestures such as tapping, scrolling, etc., is much higher and can range from 1.5 Hz to almost 20 Hz. The walking at a speed of 1 m/s produces a Doppler frequency of 50 Hz. Algorithm 1 shows the snippets of the algorithm used for performing STFT on the data for feature extraction purpose.

**Algorithm 1:** Features from FFT and STFT**Input:** I &fs **Output:** S, T, FN: Length of data (I)*Zero padding*Number of points for FFT/STFT (M): 10*N*frequency matrix*w = ([0:M-1]/M*fs) -(fs/2)*Remove DC from I*I = detrend(I)*Utilizing upper*/*lower envelopes*B = I*FFT calculation*FFT = fftshift (fft (B, M))*Human breathing*[~, I] =max (abs (FFT))Breathing rate: 60*abs(w(I))*STFT Calculation*[S, F, T] = stft (I, fs, ‘Window’,              kaiser (X*fs,5),              ‘OverlapLength’, round(Y*fs),              ‘FFTLength,’ M)
X and Y are the values used to determine window size and overlap. For vital signs X = 10 & Y = 9.5; X = 0.5 & Y = 0.1 for gestures.

**Figure 5:**
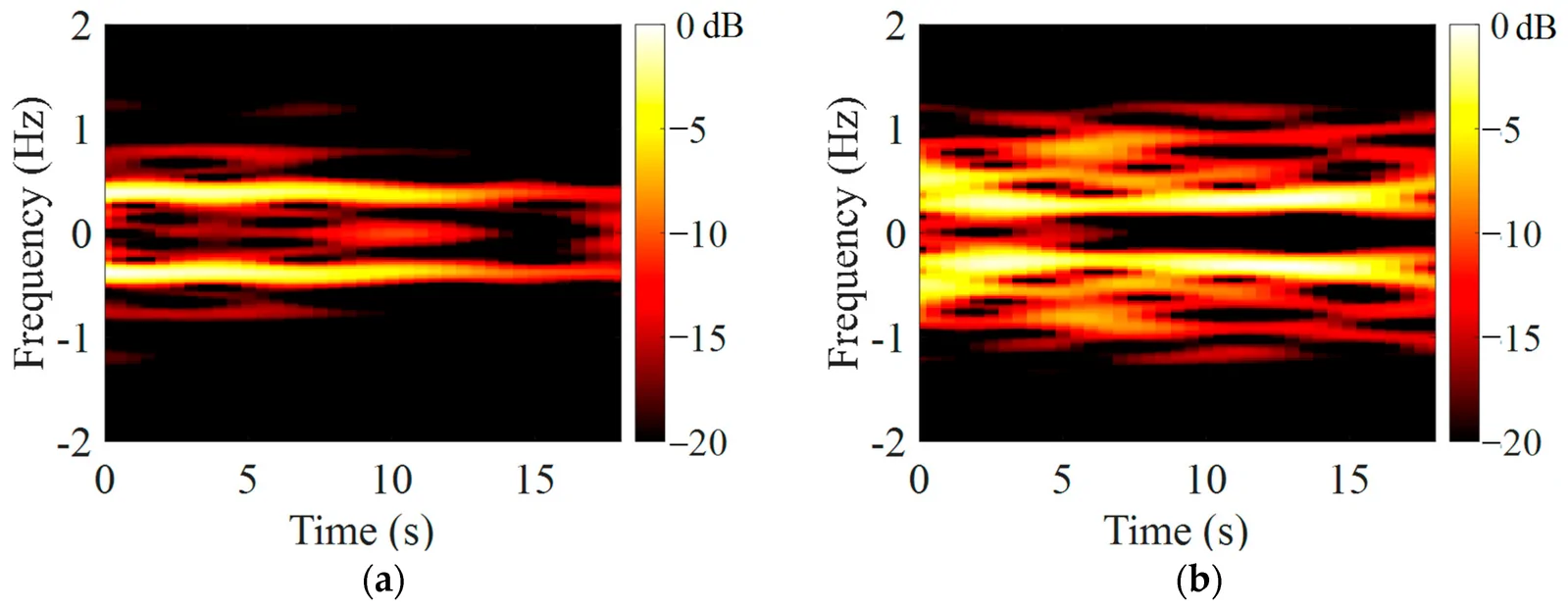
Human respiration for: (**a**) Phone on table; (**b**) phone when kept on palm.

Feature extraction: The data after pre-processing is arranged in the real form I. The phase difference between the upper and lower envelope is 180°, as can be seen from Figure 2. The feature extraction can be divided into two categories: (a) vital signs and (b) gesture. For the data processing for human vital signs, both positive and negative frequencies are shown in Figure 5 for visualization purposes, consistent with the symmetric nature of the envelope-detected signal described in Section 2. As the envelope-based method discards phase information, it does not resolve motion direction (toward versus away from the sensor); direction recovery would require phase-preserving processing such as I/Q demodulation and is identified as a direction for future work.

Vital signs: The experiments were performed in two situations: (1) phone in palm and (2) phone on the table. In both these situations, the microphone and loudspeaker of the mobile were pointing towards the human chest. The window size for vital signs was selected as 10 s to capture enough baseband data to accurately determine the breathing rate. Under normal conditions, the human breathing rate lies between 0.1 Hz and 0.4 Hz, which corresponds to 6–24 breaths per minute, which means that it takes 2.5 to 10 s to complete one breath. Hence, a window of 10 s was selected to ensure at least one breath cycle is captured.

The data captured by the microphone were pre-processed using the algorithm discussed earlier and sent to the next stage where features were extracted. Figure 5 shows the Doppler signature obtained from the received data after the preprocessing and feature-extraction stage. Respiration rate detected by the proposed system was compared against reference readings from a pulse oximeter across two subjects, with three recordings per subject of approximately 30 s in duration. Table 2 summarizes the system-detected and reference respiration rates for each subject. The mean absolute error across subjects was 0.049 Hz (SD = 0.030 Hz, 95% CI: [0.018, 0.081] Hz), with a maximum observed error of 0.09 Hz. These results are consistent with the manuscript’s earlier claim of respiration rate accuracy within 0.1 Hz of the reference, though we note the maximum error approaches this bound and the confidence interval reflects the limited sample size; validation across a larger subject pool is identified as a direction for future work.

**Figure 6:**
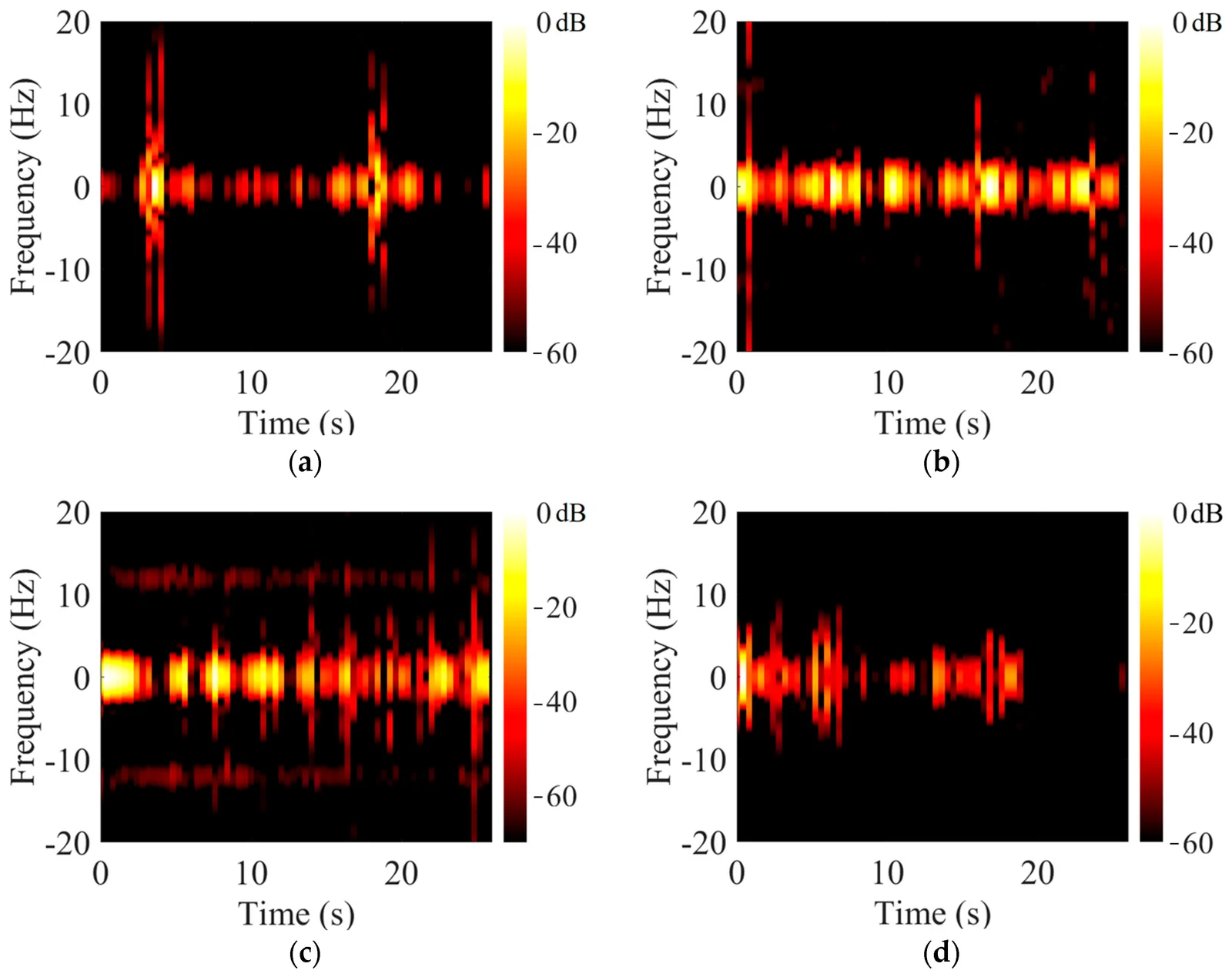
STFT plots of some gestures: (**a**) scrolling 2 times; (**b**) tapping 3 times; (**c**) talking; (**d**) typing/texting.

Gestures: The duration of gestures such as scrolling, tapping, talking, and typing over the phone is much higher than the breathing frequency. Hence, a smaller window size was utilized in this case to accurately pinpoint the occurrence of the event. Hence, a window was selected as 0.5 s with an overlap of 0.1 s. Figure 6 shows the STFT plots obtained for the gestures using a 0.5 s window size with an overlap of 0.1 s.

### 4.2. Noise and Power Computation

**Figure 7:**
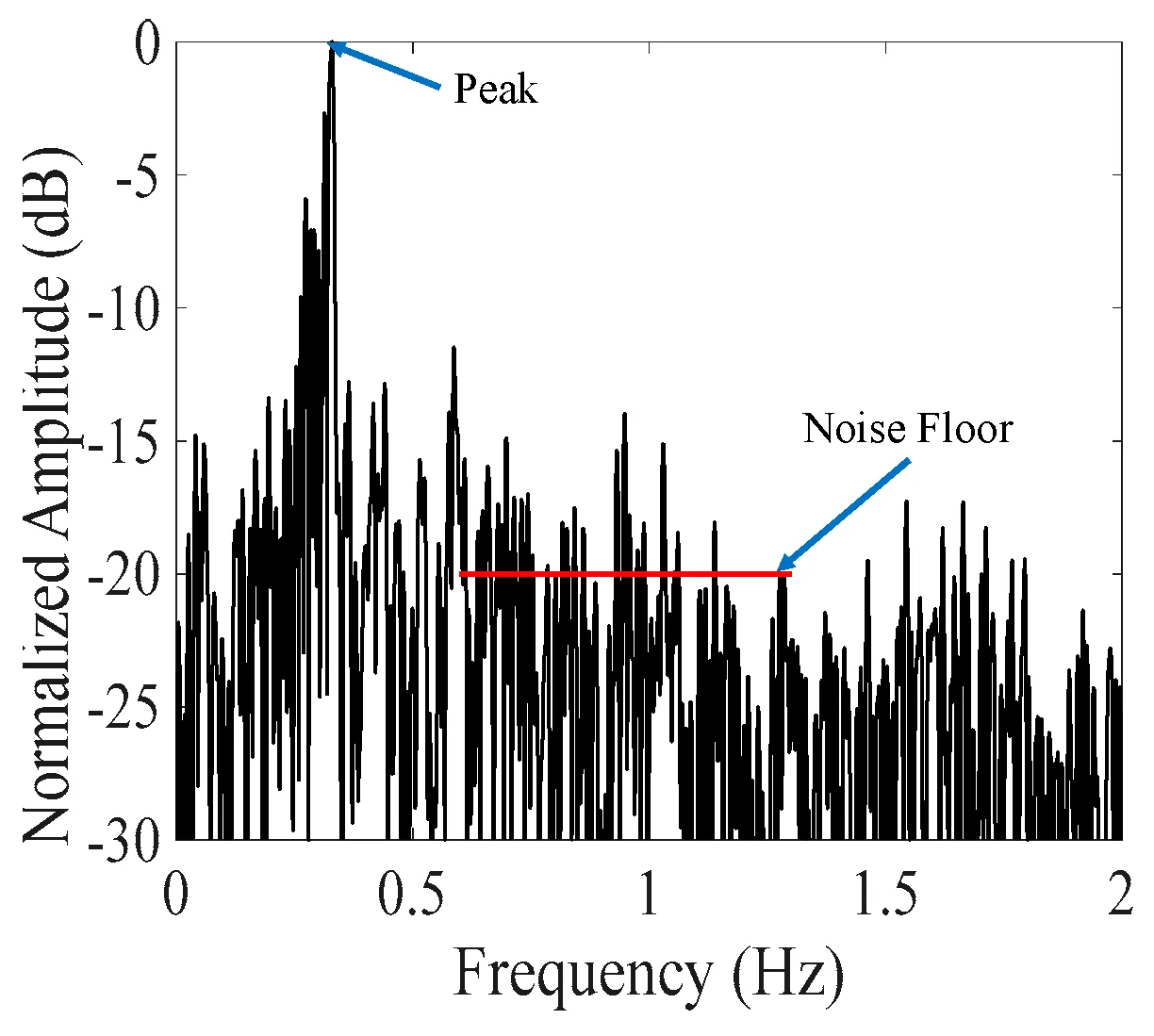
Calculation of noise floor from human respiration data.

Noise computation: An FFT was performed on the envelope signal obtained from the received signal. The FFT plot shown in Figure 7 shows the respiration rate of the human target. The amplitude was normalized and converted to a logarithmic scale. The respiration of the human lies between 0.1 Hz to 0.6 Hz, and human gestures can begin as low as 1.5 Hz. For calculating the noise floor, the region between 0.6 Hz and 1.3 Hz was utilized, chosen to remain close to the signal band, since noise in this system is not spectrally flat and a region further from the signal would underestimate the local noise floor while staying below the lowest reported gesture frequency to avoid contaminating the estimate with gesture energy. An average value of the amplitude from 0.6 Hz to 1.3 Hz was calculated, which was termed as the noise floor of the signal and was plotted in Figure 7.

### 4.3. Bandwidth Computation and Other Features

**Figure 8:**
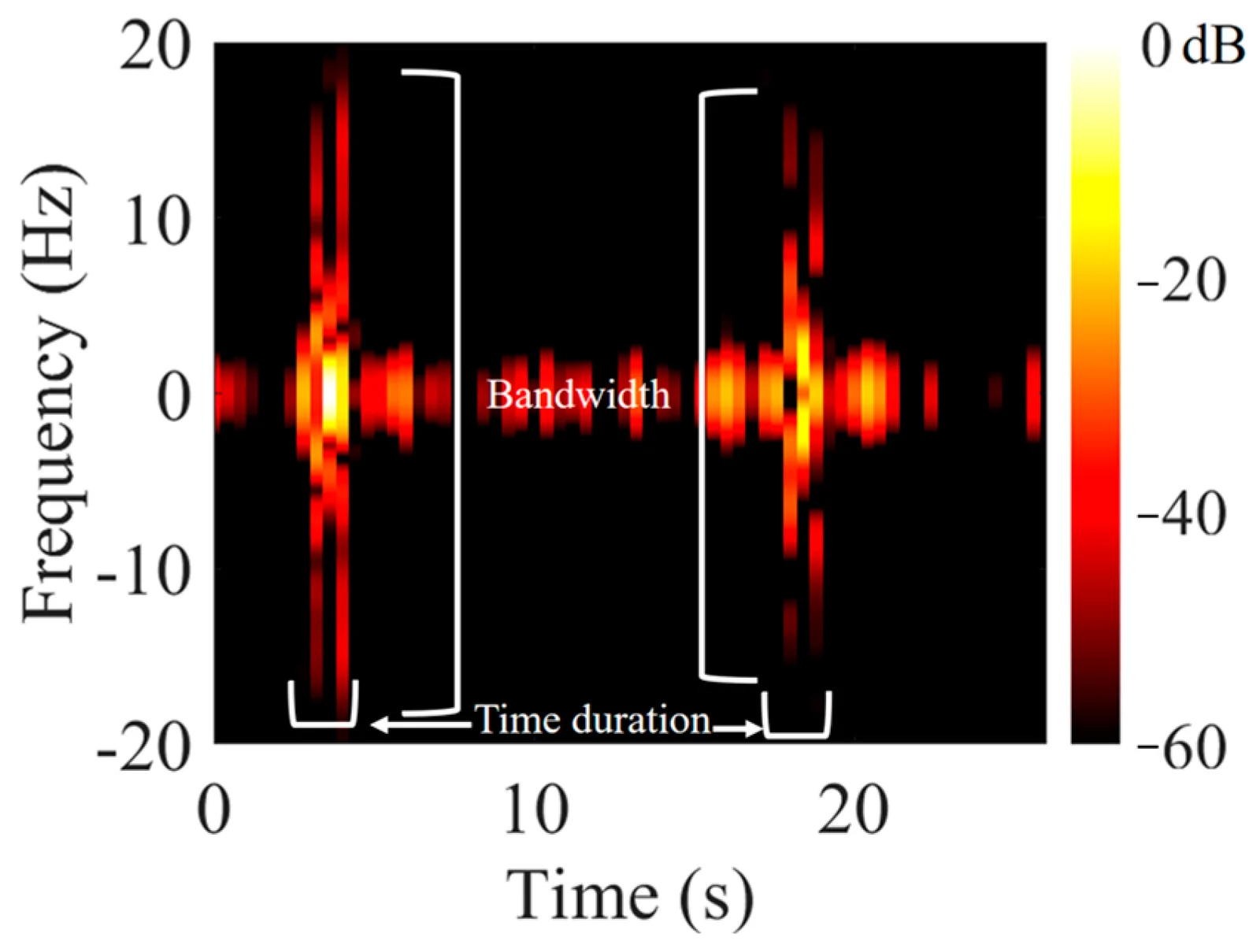
Feature extraction from STFT plot while scrolling.

The STFT for each gesture was calculated, and based on the STFT plot, and features such as bandwidth, time duration, and pattern, one gesture can be visually distinguished from another. Based on the bandwidth, time duration users were authenticated. The bandwidth here signifies the maximum and minimum frequency identified on the STFT plot. Figure 8 shows a typical STFT plot obtained from scrolling the phone. The bandwidth and time duration were identified as shown in Figure 8. The bandwidth here is approximately 36 Hz, while the time duration is 2 s. While in case of tapping, the time duration is much lower, similarly typing takes more time to complete compared to scrolling.

The gesture results presented in this work are intended as an initial feasibility demonstration, showing that different gesture types produce visually distinguishable time-frequency signatures (Figure 6 and Figure 8). Formal gesture classification is outside the scope of the present study; a machine-learning-based classification pipeline, including appropriate training/test separation and quantitative performance evaluation (accuracy, precision/recall, confusion matrix), is identified as future work, contingent on collecting a larger, multi-subject gesture dataset.

### 4.4. Effect of Window Sizes on STFT

**Figure 9:**
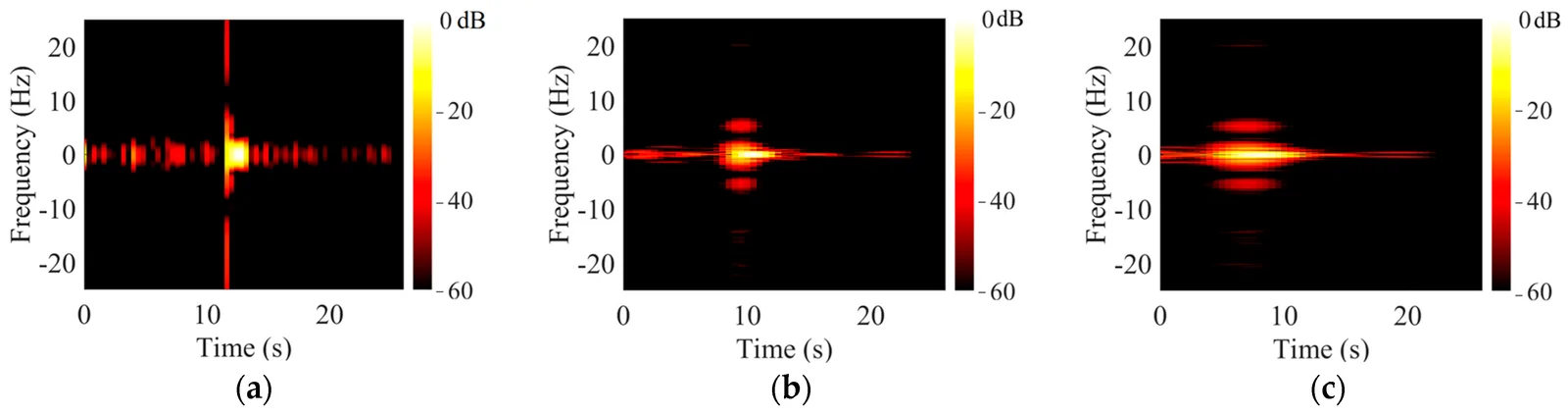
STFT plots tapping for different window sizes: (**a**) 0.5 s; (**b**) 5 s; (**c**) 10 s.

The selection of window size is important in detecting the event occurrence time. Figure 9 shows the STFT plots obtained using different window sizes. It can be seen from Figure 9 that irrespective of the window size, the event can be recorded. However, using a larger window size cannot point accurately to the event occurrence time. Also if two or more events happen in a shorter time interval, they can get merged and cannot accurately point to the number of events that happened in the time frame. For the event occurring at 12 s, Figure 9a accurately identifies its time of occurrence. A 5 s window was also able to record the event, but the plot was more spread out. Similarly, using a 10 s window spread the event further. A window size of 5 s and 10 s might not be able to distinguish between two events that occur at 1 s intervals and might report the two events as one event.

### 4.5. Phone-Specific Frequency

**Figure 10:**
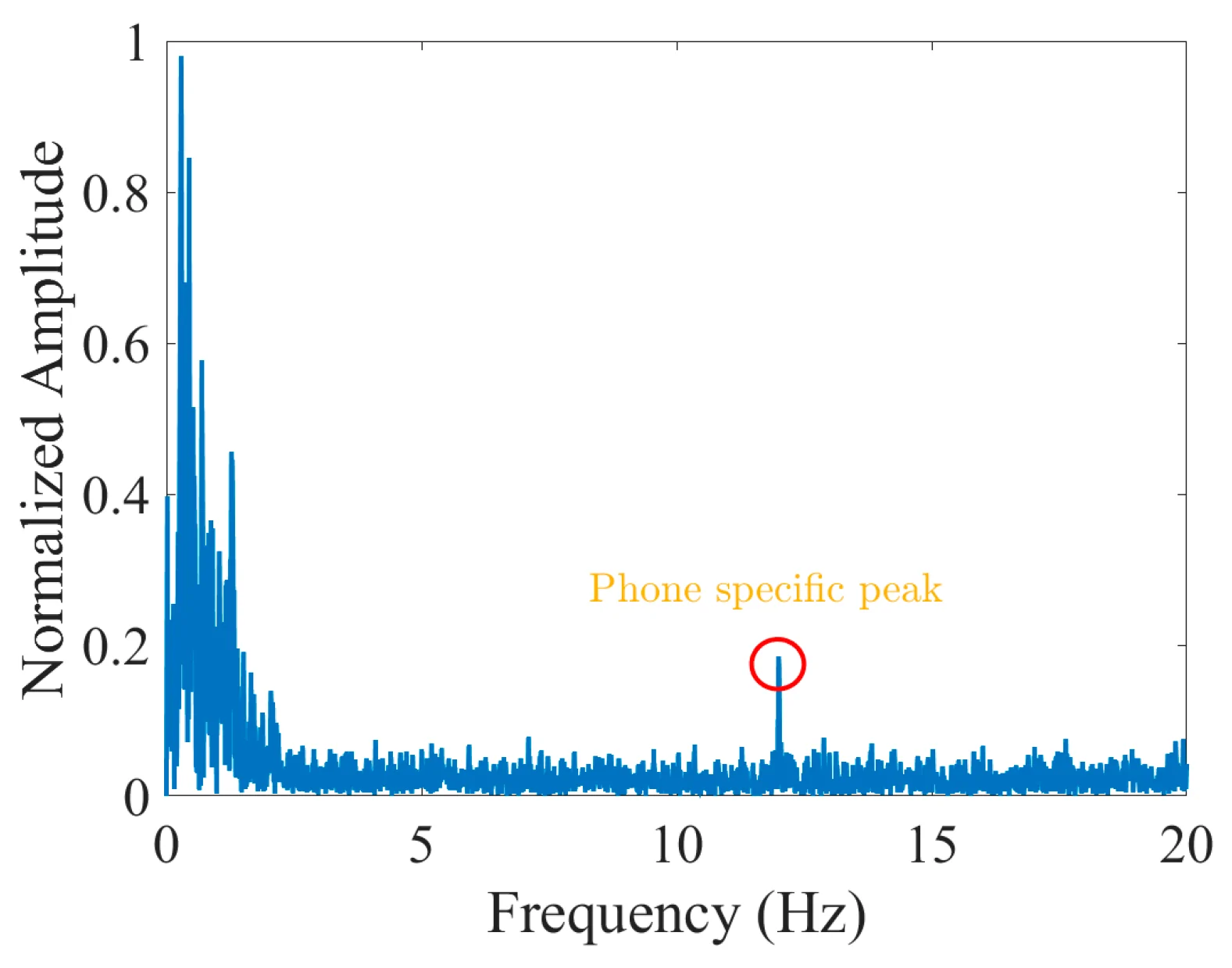
FFT plot of an envelope signal to identify phone-specific frequency.

Some phones, apart from recording the received signal, also add a constant frequency tone to the signal. The measurements indicate that this frequency lies between 11 Hz and 20 Hz, and these frequencies have a constant value for the phone. Figure 10 shows the FFT plot obtained using NUU S5702L phone for measuring respiration. As shown in Figure 10, a peak at 12 Hz is constantly present in all the experiments performed using this specific phone. This phone-specific peak can also be utilized for device authentication purposes. To assess whether this phone-specific peak generalizes across devices, the same measurement was repeated on three additional smartphone models: Samsung Galaxy S10, Samsung Galaxy S21, and Google Pixel 8. Table 3 shows the phone-specific peak frequency identified for each device. The peak was present in all four devices tested but occurred at a different frequency for each, consistent with the previously reported 11-to-20 Hz range for this artifact, confirming that it originates from device-specific hardware characteristics rather than the target motion itself, and supporting its potential use as a device signature for authentication purposes. This peak falls outside the frequency range used for respiration detection (0.1 to 0.6 Hz). It does fall within the broader frequency range associated with gestures (1.5 to 20 Hz); however, because the phone-specific peak is a constant, time-invariant tone, while gesture events appear as short, time-localized bursts in the STFT, the two are readily distinguished in the time-frequency domain. While the phone-specific peak was confirmed across four smartphone models, the core actuator and human-subject sensing experiments (motion sensitivity, respiration validation, gesture characterization) in this work were conducted using a single primary device (NUU S5702L). A full characterization of sensing performance, including sensitivity, noise floor, and signal-to-noise ratio, across multiple device models remains a direction for future work.

## 5. Conclusions

The concept of handheld sonar based on a mobile phone was successfully validated, and mm-scale motion sensitivity was demonstrated with motion amplitudes as small as 2 mm at 0.6 Hz under a controlled environment. The respiration rate detected was compared and validated with a pulse oximeter, and the error was within 0.1 Hz of the reference measurement. Without any hardware modification to a commercial phone, the solution could determine complex motion frequency i.e., motion having multiple frequency components. Since the complex motion of the target could be recorded by the sonar and extraction could be done with the envelope-based method, the proposed system can be potentially utilized for the measurement of human vital signs and monitoring patients suffering from apnea or hyperventilation. Since the proposed technology is implemented on a mobile phone, it can be conveniently utilized as a continuous health monitoring tool for mass deployment. The non-contact nature gives it an added advantage of remote monitoring without causing any inconvenience to the daily life of the users. Future work will focus on feeding the gestures and respiration signatures into a machine-learning classifier to develop a user authentication system based on individual usage patterns.

## Figures and Tables

**Table 1:** Comparison of the proposed work with state-of-the-art model.

Feature	Liu et al. [17]	Wang et al. [18]	Li et al. [19] (CW Only)	Tan et al. [21]	Proposed
Phones required	2	1	1 + laptop	1	1
Hardware modification	No	No	No	No	No
Carrier frequency	17–20 kHz	18–22 kHz	15 kHz	~19 kHz	20 kHz
Down-conversion	FFT segmentation	sin/cos multiplication	sin/cos multiplication	Bandpass + LMS filter	Envelope detection
Robust to frequency drift	No	No	No	Partial	Yes
Real-time operation	Yes	Yes	Yes	No	Near-Realtime (10 s window latency)
Application	Pedestrian tracking	Breathing only	Rotational speed	Lip reading	Vital signs + Gesture
Sensing distance	~6 m (reliable range)	Good accuracy < 50 cm, degrades sharply beyond	Tested 9–21 cm; theoretical max ~0.85 m	Not reported	~50 cm
Frequency/tracking accuracy	~2% avg. tracking error	Median error 0.2 bpm (≈0.0033 Hz); 95% <0.5 bpm	92–96% relative accuracy (RPM-based)	95.4% (mouth motion); 74.8% (sentences)	Mean absolute error 0.049 Hz
Sampling frequency	96,000 Hz	48,000 Hz	Not reported	44,100 Hz	44,100 Hz

**Table 2:** Sonar-based Vital Signs measurements.

Subject	System (Hz)	Oximeter (Hz)	Absolute Error (Hz)
S1_T1	0.266	0.25	0.016
S1_T2	0.21	0.18	0.03
S1_T3	0.23	0.28	0.05
S2_T1	0.44	0.35	0.09
S2_T2	0.43	0.35	0.08
S2_T3	0.38	0.35	0.03

**Table 3:** Comparison of phone specific frequency for different phone models.

Phone Model	Phone-Specific Peak (Hz)
NUU S5702L	12
Samsung Galaxy S10	13
Samsung Galaxy S21	14.3
Google Pixel 8	16.3

## Data Availability

Data are available upon reasonable request to the corresponding author.
